# Thiazole Antibiotics Siomycin a and Thiostrepton Inhibit the Transcriptional Activity of FOXM1

**DOI:** 10.3389/fonc.2013.00150

**Published:** 2013-06-06

**Authors:** Andrei L. Gartel

**Affiliations:** ^1^Department of Medicine, University of Illinois at ChicagoChicago, IL, USA

I read with great interest the review article by Alvarez-Fernández and Medema (2013). I was especially pleased to see that the authors comprehensively acknowledged our work by citing six of our recent papers in their review. However, when they discussed our findings on the identification of the thiazole antibiotics siomycin A and thiostrepton as inhibitors of FOXM1 transcriptional activity they made a significant mistake. They wrongly stated, “surprisingly, these inhibitors do not affect the transcriptional activity of FOXM1 *per se*.” In numerous publications we showed that indeed siomycin A and thiostrepton inhibit the transcriptional activity and the expression of FOXM1, and they also act as proteasome inhibitors (Radhakrishnan et al., 2006; Bhat et al., 2009a,b). Moreover, we demonstrated that even in the presence of high levels of exogenous FOXM1 siomycin A, thiostrepton, and known proteasome inhibitors were able to inhibit FOXM1 transcriptional activity, suggesting that this inhibition is a key initiating event for FOXM1 protein suppression (Radhakrishnan et al., 2006; Bhat et al., 2009a,b).

In addition, the authors concluded that both the mRNA and protein expression of FOXM1 are downregulated by siomycin A and thiostrepton “through an unknown mechanism” (Alvarez-Fernández and Medema, 2013). However, recently I have proposed a general model for FOXM1 inhibition by proteasome inhibitors, which links the transcriptional activity and expression of FOXM1 (Gartel, 2010, 2012). According to this model, siomycin A, thiostrepton, and other proteasome inhibitors hinder the proteasomal degradation of a negative regulator of FOXM1 (NRFM), which in return directly or indirectly inhibits the activity of FOXM1 as a transcription factor (Gartel, 2011). Because FOXM1 is involved in a positive feedback loop and activates its own transcription (Halasi and Gartel, 2009), inhibition of FOXM1 transcriptional activity leads to a decrease in its expression (Gartel, 2010). This model also anticipates that all proteasome inhibitors regardless of their structure will inhibit FOXM1 transcriptional activity through stabilizing the NRFM and subsequently (via the auto-regulatory loop) inhibit FOXM1 expression (Gartel, 2011, 2012). Overall, inhibition of FOXM1 transcriptional activity by siomycin A, thiostrepton, and other proteasome inhibitors is an essential part of their regulation of FOXM1.

FOXM1 is without a doubt emerging as a critical regulator of cancer development (Halasi and Gartel, 2013) that may affect all hallmarks of cancer (Hanahan and Weinberg, 2012). Originally, it was identified as a key regulator of cell proliferation and cell cycle progression; however, in the past few years FOXM1 became one of the central contributors to tumorigenesis. Growing body of evidence suggests that targeting this single transcription factor may have great promise for inhibiting tumor development. Great efforts are currently being undertaken to find more specific inhibitors of FOXM1 and to elucidate their mechanism of action. I believe it is essential for the scientific community to have correct information about the regulation of FOXM1 by FOXM1 inhibitors in order to progress and accelerate research in the FOXM1 field.

## References

[B1] Alvarez-Fernández M. Medema R. H. (2013). Novel functions of FoxM1: from molecular mechanisms to cancer therapy. Front. Oncol. 3:30 10.3389/fonc.2013.00030 23467617 PMC3588610

[B2] Bhat U. G. Halasi M. Gartel A. L. (2009a). Thiazole antibiotics target FoxM1 and induce apoptosis in human cancer cells. PLoS ONE 4:e5592 10.1371/journal.pone.0006593 19440351 PMC2680058

[B3] Bhat U. G. Halasi M. Gartel A. L. (2009b). FoxM1 is a general target for proteasome inhibitors. PLoS ONE 4:e6593 10.1371/journal.pone.0006593 19672316 PMC2721658

[B4] Gartel A. L. (2010). A new target for proteasome inhibitors: FoxM1. Expert Opin. Investig. Drugs 19, 235–242 10.1517/13543780903563364 20074015 PMC3532816

[B5] Gartel A. L. (2011). Thiostrepton, proteasome inhibitors and FOXM1. Cell Cycle 10, 4341–4342 10.4161/cc.10.24.18544 22134246 PMC3272263

[B6] Gartel A. L. (2012). The oncogenic transcription factor FOXM1 and anticancer therapy. Cell Cycle 11, 3341–3342 10.4161/cc.21841 22918255 PMC3466533

[B7] Halasi M. Gartel A. L. (2009). A novel mode of FoxM1 regulation: positive auto-regulatory loop. Cell Cycle 8, 1966–1967 10.4161/cc.8.12.8708 19411834

[B8] Halasi M. Gartel A. L. (2013). FOX(M1) news – it is cancer. Mol. Cancer Ther. 12, 245–254 10.1158/1535-7163.MCT-12-0712 23443798 PMC3596487

[B9] Hanahan D. Weinberg R. A. (2012). Hallmarks of cancer: the next generation. Cell 144, 646–674 10.1016/j.cell.2011.02.013 21376230

[B10] Radhakrishnan S. K. Bhat U. G. Hughes D. E. Wang I. C. Costa R. H. Gartel A. L. (2006). Identification of a chemical inhibitor of the oncogenic transcription factor forkhead box M1. Cancer Res. 66, 9731–9735 10.1158/0008-5472.CAN-06-1576 17018632

